# Total Spinal Anesthesia Following an Interscalene Block Treated with Intravenous Lipid Emulsion

**DOI:** 10.7759/cureus.4491

**Published:** 2019-04-17

**Authors:** Forest N Turner, Richard D Shih, Irina Fishman, Diane P Calello, Joshua J Solano

**Affiliations:** 1 Emergency Medicine, Charles E. Schmidt College of Medicine at Florida Atlantic University, Boca Raton, USA; 2 Anesthesiology, Good Samaritan Hospital Medical Center, West Palm Beach, USA; 3 Emergency Medicine, Rutgers New Jersey Medical School, Newark, USA; 4 Integrated Medical Science, Florida Atlantic University, Boca Raton, USA

**Keywords:** regional anaesthesia, local anesthesia, intravenous lipid emulsion

## Abstract

Total spinal anesthesia following interscalene block is a rare and life-threatening complication of regional anesthesia. A 56-year-old woman underwent an uncomplicated left shoulder bone spur removal under general anesthesia with an interscalene nerve block at an outpatient surgical center. Subsequently, she developed bilateral mydriasis, paralysis of all extremities, and respiratory arrest. She was intubated and transferred to the emergency department (ED) where she was given intravenous lipid emulsion (ILE) with complete recovery of neurological function. ILE therapy may be considered as a rescue treatment in addition to supportive therapy for total spinal anesthesia.

## Introduction

Interscalene anesthetic blocks are commonly used for providing analgesia for upper extremity procedures, especially for shoulder surgeries. Using either ultrasound or nerve stimulator guidance, local anesthetic is injected between the anterior and middle scalene muscles thereby anesthetizing the roots of the brachial plexus. Complications are rare and can include peripheral nerve damage, local anesthetic toxicity, bleeding at the site, and pneumothorax. An extremely uncommon yet potentially catastrophic complication is total spinal anesthesia resulting from the spread of the local anesthetic into the intrathecal space. While intravenous lipid emulsion (ILE) is widely used for the treatment of local anesthetic systemic toxicity (LAST), its role as a resuscitative drug in other settings such as inadvertent total spinal anesthesia remains unclear. We report a case of total spinal paralysis following a routine brachial plexus nerve block that was treated with ILE with subsequent complete recovery. This case report represents one of the first cases where ILE has been used to reverse the effects of local anesthesia causing total spinal anesthesia.

## Case presentation

A 56-year-old woman underwent an uncomplicated left shoulder bone spur removal under general anesthesia with 2 mg of versed, 100 mcg of fentanyl, 150 mg of propofol, and sevoflurane in an outpatient surgery center. After completion of the procedure, the anesthesiologist performed an interscalene nerve block for post-operative pain control utilizing 30 mL of bupivacaine (0.25%). Approximately 5 min after completion of the block, the patient developed bilateral mydriasis, paralysis of all extremities, and respiratory arrest. The patient was immediately intubated, administered IV fluids, ephedrine 15 mg IV, and transferred to an emergency department (ED). 

On ED arrival the patient was being ventilated through an oral endotracheal tube and was completely paralyzed. Her vital signs were: blood pressure (BP) 108/56 mmHg; pulse 86 per minute; respiratory rate 24 breaths per minute on a ventilator. Her pupils were 6 mm and unresponsive to light bilaterally. She had no response to painful stimulation and had no spontaneous respirations on a ventilator. Her initial blood tests included a complete blood count, electrolytes, liver function tests, cardiac enzymes, and coagulation tests. The lab results were all unremarkable except for a phosphorus of 1.7 mg/dL and lactate of 4 mmol/L. In the ED she was administered a 1-L normal saline bolus and 20% intravenous lipid emulsion 85 mg. Over the next 4 h, the patient progressively regained motor and sensory functions followed by successful extubation in the ED. She was admitted to the hospital for observation and discharged home the next day without any neurologic sequelae.

## Discussion

Interscalene (brachial plexus) anesthetic blocks are commonly used to provide analgesia both intraoperatively and postoperatively for upper extremity surgical procedures. Local anesthetic is injected in the interscalene groove between the anterior and middle scalene into the brachial plexus at the level of C6. The nerve block is performed either with ultrasound technique with visualization of the nerve roots or via landmark technique and a nerve stimulator. Paresthesias typically extend from the shoulder down the radial aspect of the arm, while usually sparing the ulnar aspect of the arm and hand [1].

Complications from this regional block are rare, especially when performed by experienced anesthesiologists [2]. A study of 3,172 blocks estimated the complication rate at 1.1%. These complications included peripheral nerve damage both persistent and recoverable, local anesthetic toxicity manifesting as either cardiovascular events or seizures, phrenic nerve palsy, pneumothorax, and total spinal paralysis [2]. In the four cases reported by Scammell et al. patients quickly developed total spinal paralysis after injection of the block but spontaneously recovered over several hours with cardiorespiratory support [3-6]. In four cases reported by Benumof, another by Mostafa, and one by Barutell, spinal block was followed by permanent neurologic deficits of varying degrees as well as evidence of spinal cord damage on MRI [7-9]. In the only other case where ILE was used, spinal anesthesia resolved spontaneously with no sequelae [10].

Total spinal paralysis involves inappropriate introduction of local anesthetic into the subarachnoid space and is characterized by apnea, dilated pupils, absent corneal reflexes, and hypotension [11]. As this complication is extremely rare, there is scarce information to guide treatment beyond the few previously reported cases. Many of the reported cases follow a similar series of events: total spinal anesthesia during infusion of anesthetic or shortly thereafter; intervention with cardiorespiratory support; and spontaneous recovery over the course of several hours. Metabolism of bupivacaine and subsequent spontaneous recovery is expected over several hours, as the half-life of bupivacaine is approximately 50 min, but highly variable [12]. Conversely, several of the reported cases with a similar presentation to ours demonstrate total spinal paralysis initially with partial recovery associated with permanent or long-term neurologic sequelae.

The cause for this unusual complication of total spinal anesthesia is not entirely clear. Proposed mechanisms include intraneural injection and spreading of local anesthesia centrally, injection directly into the intrathecal space, or injection into a lateral projection of the dural sac outside of the intervertebral foramen known as a “dural cuff” anatomical variation [6]. Furthermore, it is unclear why some cases are associated with permanent neurologic deficits. Of note in cases where a total spinal block developed, aspiration during the injection throughout the performance of the block was negative for any intrathecal fluid.

Intravenous lipid emulsion is used for the treatment of severe local anesthetic toxicity especially in the setting of cardiovascular collapse [13]. One of the main purported mechanisms for ILE’s antidotal benefits in these cases has been theorized to be due to a “lipid sink.” When intravascular lipid is administered it may bind to a lipophilic toxin such as a local anesthetic, thereby reducing the available drug in target tissues such as the brain and myocardium. Complications associated with the use of ILE therapy for local anesthetic toxicity are rare, but include interference with laboratory testing, pancreatitis, acute respiratory distress syndrome, fat overload syndrome, hypertriglyceridemia, and deep vein thrombosis [14-15].

An evolving area of investigation regarding ILE involves its use for alternate indications including the treatment of total spinal anesthesia and prolonged neural blockade. Gupta reported on a 43-year-old male presenting to the ED after developing respiratory arrest following an interscalene block. The patient was intubated prior to transfer to the ED. In the ED he was first hemodynamically stabilized with fluids and dopamine and then given ILE therapy. He regained brainstem reflexes 30 min following this treatment with a full recovery 1 h afterwards [16]. Eldor reports on three cases of women receiving spinal anesthesia that progressed to high spinals who were treated with ILE and showed a recovery shortly thereafter suggesting a temporal relationship with ILE administration [17]. Apart from its role in resuscitation following accidental development of total spinal anesthesia, ILE has been used to reverse undesired effects of local anesthesia. Elder reports of a case of a 71-year-old woman who received a combined spinal epidural for a total knee replacement who was complaining 8 h post-operatively of diminished sensory and motor sensation on the nonoperative lower extremity. She was given ILE and 60 min later she recovered sensation and motor strength of her lower extremities [18].

In our case, the patient recovered completely over 4 h. It is difficult to ascertain whether administration of ILE contributed to her recovery. As bupivacaine and other local anesthetics can cross the blood-brain barrier, the use of ILE would theoretically speed recovery by potentially drawing the local anesthetic out of the CSF. However, several cases document spontaneous recovery in a similar timeframe as our case without utilizing ILE therapy. Those cases were treated exclusively with supportive care including intubation, mechanical ventilation, vasopressors, antidysrhythmics, fluids, and sedation [3-6]. Additionally, it is unclear if ILE therapy would reduce the poor neurologic outcomes of some of the cases. Several of these cases revealed a syrinx, a fluid-filled cyst within the spinal cord, on MRI suggesting damage from direct local anesthetic injection into the spinal cord [7-8].

## Conclusions

Total spinal anesthesia from interscalene local anesthetic block is an uncommon yet potentially catastrophic complication. ILE may be beneficial to reverse the clinical syndrome and hasten recovery. Several case reports along with ours demonstrate favorable outcomes following the administration of ILE for total spinal anesthesia. These findings highlight the need to further explore the use of ILE to reverse this and other adverse effects of local anesthetics.
